# Retraction Note: MiR-125 family improves the radiosensitivity of head and neck squamous cell carcinoma

**DOI:** 10.1007/s11033-024-09283-1

**Published:** 2024-01-30

**Authors:** Qi-Wei Wang, Ya-Nan Sun, Li-Jun Tan, Jian-Nan Zhao, Xiao-Jie Zhou, Tian-Jiao Yu, Jiang-Tao Liu

**Affiliations:** 1 Department of Otolaryngology, Head and Neck Surgery, Harbin Medical University, Harbin, People’s Republic of China; 2 Department of Otolaryngology, Head and Neck Surgery, The Second Affiliated Hospital of Harbin Medical University, No.246, Xuefu Road, Harbin, 150081 People’s Republic of China; 3 Department of Oncology, The First Affiliated Hospital of Harbin Medical University, Harbin, People’s Republic of China; 4 Department of Otolaryngology, Head and Neck Surgery, The First Affiliated Hospital of Harbin Medical University, No.23, Youzheng Street, Harbin, 150001 People’s Republic of China

**Retraction Note: Molecular Biology Reports (2023) 50:5307–5317**

10.1007/s11033-023-08364-x

The Editor-in-Chief has retracted this article after concerns were raised about the data presented. The paper reports the use of the Hep-2 cell line as a model of laryngeal cancer cells. After publication, the journal was notified that this cell line is contaminated with the HeLa cell line and in fact contains cervical adenocarcinoma cells. The Editor-in-Chief therefore no longer has confidence in the reliability of the conclusions. Ya-Nan Sun did not explicitly state whether or not they agree with this retraction. All other authors did not respond to correspondence from the publisher about this retraction.

